# Comparison of Potato and Asian Citrus Psyllid Adult and Nymph Transcriptomes Identified Vector Transcripts with Potential Involvement in Circulative, Propagative Liberibacter Transmission

**DOI:** 10.3390/pathogens3040875

**Published:** 2014-11-03

**Authors:** Tonja W. Fisher, Meenal Vyas, Ruifeng He, William Nelson, Joseph M. Cicero, Mark Willer, Ryan Kim, Robin Kramer, Greg A. May, John A. Crow, Carol A. Soderlund, David R. Gang, Judith K. Brown

**Affiliations:** 1 School of Plant Sciences, The University of Arizona, Tucson, AZ 85721, USA; E-Mails: twfisher@email.arizona.edu (T.W.F.); molupapa@gmail.com (M.V.); jmc6@ag.arizona.edu (J.M.C.); 2 Institute of Biological Chemistry, Washington State University, Pullman, WA 99164, USA; E-Mails: rfhe@wsu.edu (R.H.); gangd@wsu.edu (D.R.G.); 3 BIO5, The University of Arizona, Tucson, AZ 85721, USA; E-Mails: will@agcol.arizona.edu (W.N.); mark@agcol.arizona.edu (M.W.); cari@agcol.arizona.edu (C.A.S.); 4 National Center for Genome Resources, 2935 Rodeo Park Drive East, Santa Fe, NM 87505, USA; E-Mails: rwkim@ucdavis.edu (R.K.); robin.kramer@nih.gov (R.K.); gregory.may@pioneer.com (G.A.M.); john.crow@pioneer.com (J.A.C.)

**Keywords:** circulative-propagative transmission, fastidious plant bacteria, psyllid vector, transcriptome

## Abstract

The potato psyllid (PoP) *Bactericera cockerelli* (Sulc) and Asian citrus psyllid (ACP) *Diaphorina citri* Kuwayama are the insect vectors of the fastidious plant pathogen, *Candidatus* Liberibacter solanacearum (CLso) and *Ca*. L. asiaticus (CLas), respectively. CLso causes Zebra chip disease of potato and vein-greening in solanaceous species, whereas, CLas causes citrus greening disease. The reliance on insecticides for vector management to reduce pathogen transmission has increased interest in alternative approaches, including RNA interference to abate expression of genes essential for psyllid-mediated *Ca.* Liberibacter transmission. To identify genes with significantly altered expression at different life stages and conditions of CLso/CLas infection, cDNA libraries were constructed for CLso-infected and -uninfected PoP adults and nymphal instars. Illumina sequencing produced 199,081,451 reads that were assembled into 82,224 unique transcripts. PoP and the analogous transcripts from ACP adult and nymphs reported elsewhere were annotated, organized into functional gene groups using the Gene Ontology classification system, and analyzed for differential *in silico* expression. Expression profiles revealed vector life stage differences and differential gene expression associated with Liberibacter infection of the psyllid host, including invasion, immune system modulation, nutrition, and development.

## 1. Introduction

The potato/tomato psyllid (PoP), *Bactericera cockerelli* (Sulc) (Triozidae) is a hemipteran insect that colonizes mainly plant species in Convolvulaceae and Solanaceae. PoP transmits a recently discovered, fastidious bacterium that has been associated with the emergence of the plant diseases, zebra chip of potato (affecting leaves and tubers) and vein greening of tomato (affecting leaves and fruit) and other solanaceous species [1,2,3]*.* The causal agent is the phloem-limited bacterium, *Candidatus* Liberibacter solanacearum (CLso), also known as *Ca*. L. psyllaurous [4] that is transmitted by *B. cockerelli* [5]. Until recently, the potato psyllid was known only for its association with “psyllid yellows” disease of tomato, pepper, and potato plants, syndromes attributed to psyllid feeding alone and to a salivary toxin [6]. These observations strongly suggest that CLso emergence and PoP transmission of it is a recent phenomenon. In contrast, the Asian citrus psyllid (ACP), *Diaphorina citri* is well known as the vector of *Ca*. L. *americanus*, *asiaticus*, and *africanus,* also fastidious bacteria and the causal agents of citrus greening disease, or Huanglongbing (HLB) [7,8]. HLB has reached a crisis-level in the citrus industry worldwide [9].

The genus *Ca*. Liberibacter contains obligate, phloem-limited gram-negative bacteria classified in the α-Proteobacteria [10]. Both CLas and CLso multiply in both the plant and their psyllid host, and therefore utilize a circulative-propagative mode of transmission [11,12,13,14,15]. Evidence that CLas is detectable in ACP eggs by polymerase chain reaction (PCR) suggests a low level of transovarial transmission [4,16], however, additional evidence is needed for a direct role in the life cycle to confirm this observation. Also, transmission from ACP males to females has been reported based on qPCR detection in eggs that were dissected from uninfected females following mating with CLas-infected males [17]. The low frequency of CLas detection in ACP eggs and sexually, at 2%–6%, suggests that vertical transmission may be an important as a survival mechanism when a suitable CLas plant host is not available. Transmission electron microscopy (TEM) studies have revealed lesion-like pores on the external surfaces of CLso-infected PoP guts [18] that when taken together with reduced fecundity and nymphal survival for CLso-infected PoP [19,20], suggests CLso and perhaps other *Ca*. Liberibacter have evolved a parasitic relationship with their psyllid vector.

ACP and PoP harbor endosymbiotic bacteria, including the primary and secondary endosymbionts, *Carsonella ruddii* [21,22] and *Arsenophonus*, respectively, both α-Proteobacteria. In addition, *Wolbachia* sp., a parasitic bacterium most well known in arthropods for perturbing host reproduction [23], was detected in all nymphal and adult PoP life stages [22], and infecting 76.2% of ACP adults [24]. Psyllid defense responses, particularly those that counter invasion and nutritional deprivation [25], may exhibit species-specific host-parasite relationships, some which may depend on the duration of parasite-host exposure, and different interactions they have evolved with their endosymbionts [26]. Certain ACP endosymbionts require nitrogenous waste recycling and partitioning of other resources associated with ecological adaptations [27,28,29], a relationship that is mutually beneficial to them and their psyllid host [28,29,30]. In contrast, CLas and CLso, which propagate and circulate in their psyllid host, may perturb the host innate immunity and other stress responses, while also exploiting host nutritional stores required for colonization, multiplication, circulation, acquisition, and transmission to the plant host.

Transmission efficiency of CLas and CLso by ACP and PoP, respectively, appears to be dependent on the life stage of the vector during ingestion, and possibly also during the acquisition phase [11,16,31]. Transmission of CLas by ACP adults has been shown to be most efficient when the bacterium is ingested during the nymphal stages, compared to adults [11,16]. This observation is supported by evidence that 40% of adult ACP given a 5-wk acquisition access period (AAP) on CLas-infected plants were positive for CLas ingestion, and yet when given an inoculation access period (IAP) to susceptible citrus seedlings, were unable to transmit the bacterium [16]. In contrast, 60% of ACP adults that ingested CLas when reared from the egg through the adult stage on CLas-infected plants transmitted the bacterium 73% of the time, when provided a 30-d IAP. This supports the observation that CLas multiplication in the nymphal stages is essential for efficient adult-mediated transmission [11] but it does not rule out the possibility that the primary barrier to adult transmission is an insufficient titer of CLas owing to ingested during adult instead of nymphal stages, and not an anatomical one.

In contrast, differences in CLso transmission frequency as a result of PoP life stage indicate that adult transmission between potato plants is 30% higher when psyllids are reared from egg to adult stage on CLso-infected plants, in comparison to nymphs when similarly reared on CLso-infected plants, despite each having a 7 d-IAP [31]. In another study, the 1st and 2nd PoP instars were found to have a lower CLso titer than the “older” 4th–5th nymphal stages or adults, observations that were supported by two respectively distinctive transcript profiles [32].

Similarly, taking into account clues from plant host responses to PoP feeding, gene expression studies in tomato plants colonized by CLso-infected PoP adults or nymphs showed that host gene expression was greatly affected by the particular life stage of CLso-infected psyllids, in that colonization by CLso-infected nymphs, compared to CLso-infected adults, resulted in the production of more differentially expressed plant transcripts [33]. Lastly, plant defense pathways known to be regulated by jasmonic acid and salicylic acid were suppressed in tomato plants exposed to the “older” nymphs and adults compared to 1st and 2nd nymphal instars.

Management of Liberibacter-incited diseases currently relies on insecticides to reduce the psyllid vector population and thereby the frequency of *Ca*. Liberibacter transmission [34,35]. The transmission cycle of *Ca*. Liberibacter in the psyllid is poorly studied. Only recently have studies suggested that *Ca.* Liberibacter multiplies both in the plant and psyllid vector [12,13,14,15,16]. Understanding of the CLso and CLas circulative, propagative transmission pathway at the molecular, cellular, and functional genomics levels is of interest to enable the discovery of psyllid-Liberibacter interactions that can be targeted using RNA interference (RNAi) technology [36,37] or protein overexpression [38] to abate interactions essential for Liberibacter invasion, propagation, and transmission by the psyllid host.

The objective of this study is to determine gene expression profiles and use them to infer (predict) the basis for biological inference, based on *in silico* annotation of the differentially expressed genes of PoP and ACP adults and nymphs, reared from the egg to adult stages on CLas- and CLso-infected plants. A particular focus is the identification of differentially expressed psyllid genes predicted to be essential for direct or indirect interactions with Liberibacter effectors required for CLso and CLas to establish a circulative, propagative relationship with their PoP and ACP vector, respectively.

The transcript libraries produced for PoP psyllid adults and nymphs known to be infected with, or free of, CLso were sequenced using Next Generation Sequencing (NGS) Illumina technology and assembled into 82,224 *B. cockerelli* transcripts. Transcript comparisons were carried out using the Transcriptome Computation Workbench [39] and OrthoMCL software with an option for clustering. Using this approach it was possible to select a suite of differentially expressed contigs with a high degree of confidence from among the PoP and ACP (45,976) Illumina transcripts [40]. All databases have been made available with the TCW Java software, which can be queried at the website using the URL http://www.sohomoptera.org/ACPPoP.

A large number of up or down regulated psyllid genes (contigs) were identified and functionally annotated *in silico* using all available invertebrate (including insect), bacterial, viral databases.Among these a number of contigs of interest were mined, and found to have potential involvement in *Ca*. Liberibacter infection, adhesion, multiplication, and biofilm formation, as well as in host-parasite nutrient partitioning in the gut, circulation of Liberibacter in the hemolymph, and in entry into and acquisition by the salivary glands. The gut, hemolymph, and salivary glands have been implicated in CLas and CLso infection and propagation (entry, adhesion, multiplication), circulation, and association with the salivary gland region based on TEM and light microscopic localization studies [12,13,14,15,16,18,41] and validation by quantitative PCR detection [13,14,15,16,42]. Also, because the structure of the alimentary canal of ACP and PoP adults were found to be highly similar, it is considered likely that the circulative, propagative route of *Ca.* Liberibacter in anatomical context would be similar for both vectors, ACP and PoP [43]. In part, the functional genomics results supported this general hypothesis, while at the same time, despite conserved anatomical homologies, ACP and PoP adults and nymphs were found to respond differently to infection by their particular *Ca*. Liberibacter parasite. This was particularly striking for a set of genes with predicted functions in adhesion and immunity that were found to be differentially expressed in ACP nymphs but not particularly activated in the adults. In contrast, CLso invasion of PoP resulted in greatly perturbed gene expression in adults compared to nymphs. Thus, the parasitic strategies of these *Ca.* Liberibacter species appear to be uniquely related to the particular Liberibacter-psyllid complex. These differences may be found to be indicative of additional stage-specific characteristics, including the distinct transmission phenotypes observed for ACP and PoP adults and nymphs, respectively.

## 2. Results and Discussion

### 2.1. Assembly and Annotation of Potato Psyllid Transcriptomes

**Figure 1:**
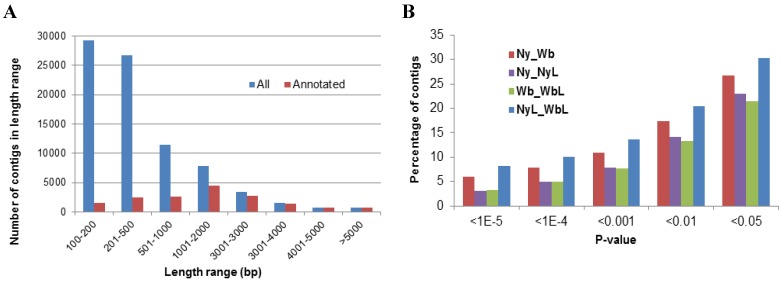
(**A**) The distribution of the lengths of potato psyllid contigs. The 199,081,451 high-quality Illumina reads determined from *Ca*. Liberibacter solanacearum-infected and uninfected adult or nymphal stage of the potato psyllid were assembled to yield 81,682 contigs, with a total size of 53.4 Mb, and average length of 654 base pairs (bp) that ranged in length from 100 to 27,405 bp; (**B**) The percentage of differentially expressed potato psyllid contigs among the different libraries and treatments at different *p*-values, based on EdgeR statistical analysis.

Four Illumina paired-end libraries were constructed for four treatments: whole bodies of the uninfected potato psyllid adults (Wb); whole bodies of *Liberibacter*-infected potato psyllid adults (WbL), uninfected potato psyllid nymphs (Ny), and *Liberibacter*-infected potato psyllid nymphs (NyL). Each library was sequenced in a single lane in an Illumina flow-cell using the Genome Analyzer IIx platform (Illumina, San Diego, CA, USA) to generate 2 × 54 bp independent reads from either end of a 250 ± 25 bp insert library fragment, resulting in a total of 199,081,451 clean reads, including 46,681,564 reads from library Wb, 53,240,863 reads from library WbL, 43,322,502 reads from library Ny and 55,836,522 reads from library NyL (Supplementary Table S1). High-quality reads were assembled to produce 82,224 contigs, 53.4 Mb in size, with an average length of 651 bp and distribution ranging from 100 to 27,405 bp in length: at 64% > 200 bp, 31% > 500 bp and 17% >1000 bp (Figure 1A). The average GC content of contigs was 40.6% and ranged from 12.1% to 82.4%. Contigs averaged 2421 sequencing reads (total expression level) and 17 reads per kilobase per million reads (RPKM). Within the TCW, the edgeR statistical software [44] was executed and the results entered into a database for query. More than 20% of contigs were significantly, differentially expressed, using a *p*-value cutoff of 0.05. The majority of differences were between the NyL × WbL and Ny × Wb comparisons, having the greatest number of differentially, expressed contigs (Figure 1B).

Of the 82,224 PoP contigs, 16,762 (20%) were annotated *in silico* to produce 28.9 Mb of sequence data, with N50 [45] and average lengths of 1390 bp and 1756 bp, respectively. The annotated contigs ranged in size from 100 to 27,405 bp with 91% > 200 bp, 76% > 500 bp, and 60% > 1000 bp (Figure 1A). Expression of the total annotated contigs comprised of 8212 sequencing reads (total) and 33 RPKM (Supplementary Table S1).

Most annotated PoP and ACP contigs [40] shared their greatest homology with sequences in the invertebrate UniProt database [46,47]. The potato psyllid (PoP) transcriptome contained more reads, at 199.1 million compared to the ACP transcriptome (129.6 million) and had a larger assembly size (53.4 Mb *vs.* 50.7 Mb) and more transcripts (82,224 *vs*. 45,976). But, PoP contigs were overall shorter on average, at 651 bp compared to ACP at 1106 bp. The lower percent of annotated PoP contigs at 20%, compared to 30% for ACP could suggest true intraspecific genomic differences between the two psyllids species, or it could possibly be due to differences in the average length of the contigs, even though the RNA quality was comparable for both.

**Figure 2:**
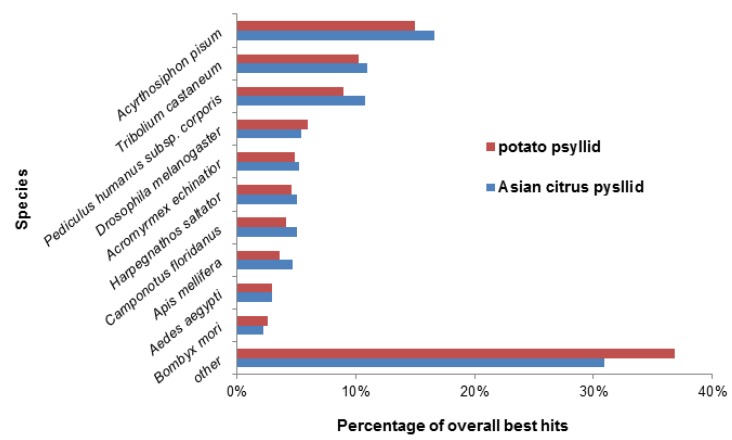
Species distribution of annotated contigs of adults and nymphs of the potato psyllid and Asian citrus psyllid. Data represent species of the first best hit obtained when contigs were blasted against the Swiss-Prot and Translated European Molecular Biology Laboratory (TrEMBL) protein databases for bacteria, invertebrates, and viruses. The majority of the annotated contigs matched to invertebrate-like proteins. The potato and Asian citrus psyllids shared similar overall homologies with the annotated contigs available for other insect species. The highest percentage of matches was to the pea aphid (*Acyrthosiphon pisum*).

Based on species-level homology, ACP and PoP showed a similar trend (Figure 2) in percentage of contigs with matches to pea aphid (*Acyrthosiphon pisum*) genes, at 15% for PoP and 17% for ACP. The next closest shared homology with other insects was in descending order for PoP and ACP, respectively, the red flour beetle (*Tribolium castaneum*) at 10% and 11%, body louse (*Pediculus humanus corporis*) at 9% and 11%, and fruit fly (*Drosophila melanogaster*) at 6% and 5%.

Most of the potato psyllid contigs (49%) annotated by bacterial databases available in SwissProt or TrEMBL were found to represent *Wolbachia* (GI:58535449) transcripts, and the remainder, approximately 18%, were mapped to the CLso genome (GI:313495152) sequence. The bacterial transcripts are thought to have been co-isolated with PoP transcripts. This is consistent with CLso presence in PoP and with previous knowledge of PoP infection by *Wolbachia* [22]. Manipulation of this naturally occurring *Wolbachia* sp. could possibly provide a vehicle for transgene delivery to the psyllid host [48].

Nachappa *et al.* [22] associated the bacterial species *Ca.* Carsonella ruddii, *Wolbachia*, CLso, *Acinetobacter*, and *Methylibium* with CLso-infected and -uninfected potato psyllids. The (contaminating) bacterial contigs reflected in our dataset shared no similarity to *Acinetobacter* or *Methylibium* transcripts. However 2% were from the primary endosymbiont, *Ca.* C. ruddii [49]. In addition, the bacterial contigs matching CLso genes were found only in the CLso-infected PoP libraries and comprised 84% of the total bacterial transcriptome. Even though these bacterial transcripts were unintentionally co-isolated with psyllid transcripts, it is apparent that not all five bacteria harbored by PoP were represented in the final dataset. This is possible due to their differential localization in psyllid tissues and organs, some of which were not specifically enriched for in library construction, and/or perhaps to differences in transcript copy number.

**Figure 3:**
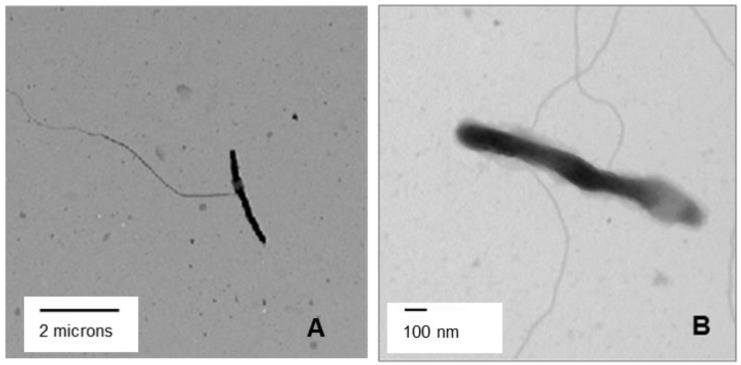
Negative-stained transmission electron micrograph from *Candidatus* Liberibacter solanacearum (CLso)-infected potato psyllid (PoP) hemolymph showing putative pilus- or flagella-like appendages of CLso.

Among the CLso contigs, the most interesting with respect to adhesion, invasion-virulence, and potential motility were the pilus- and flagellar-related genes, and further of interest was that they were expressed at levels sufficient to detect them. The genome sequences of CLso and CLas (GI: 342316098) are known to encode these genes [8,50], and the CLas flagellin gene has been shown to be expressed as a functional protein [51]. Here, we used TEM to visualize long, strand-like appendages, sometimes alone or in multiples, emanating from the sides of some CLso cells on negative-stained grids (Figure 3). The appendages averaged 4 μm in length and were strikingly similar in appearance to the flagella and/or pilus-like structures of *Ralstonia solanacearum* reported by Wairuri *et al.* [52]. Also, the expression has been validated for several flagellar and pilus-like bacterial transcripts that were assembled with PoP transcript libraries by reverse transcriptase PCR (RT-PCR) [41; JX629450 and JX629449]. These transcripts were expressed only in CLso-infected psyllids suggesting they are encoded in the *Ca*. Liberibacter genome. Interference with expression of flagellar- or pilus-like genes could Liberibacter motility and/or adhesion of CLso/CLas within the psyllid, and possibly, in the host plant as well.

Seven additional psyllid contigs were annotated as a ferroxidase-like enzyme (E-values, of E-12 to E-78), feasibly functioning in iron metabolism. Up-regulation of this psyllid enzyme in response to CLso infection could indicate induction by a CLso virulence factor(s) [53]. The CLso genome contains no annotated ferroxidase-coding regions, indicating the psyllid host may provide the CLso requirements for this enzymatic function. Down-regulation of a psyllid gene such as the latter seems strikingly essential for iron-metabolism in Liberibacter, and so could reduce iron availability that would result in starvation of the host, inadvertently undermining CLas/CLso multiplication. Thus, the effect of down-regulating the enzyme also could be detrimental to psyllid host survivability.

A large number of contigs (6.3%) were annotated using the viral sequence database in SwissProt and TrEMBL that shared homology with phage- and non-phage-like proteins. Several non-phage matches were found to the *Nuclear polyhedrosis virus* (NPV) and to densovirus-like sequences. Several NPV-related genes shared homology with cathepsin and an ecdysteroid UDP-glucosyltransferase, both genes whose expression could be detrimental to psyllid tissue integrity and potentially support CLas infection [54,55].

### 2.2. Effects of Potato Psyllid Adult and Nymph Life Stages, and Liberibacter Infection on Gene Expression

Psyllids are hemimetabolous insects and have morphologically distinct juvenile and adult stages. Both vector age and lifespan have been shown to be determinants of transmission efficiency [56], and *Ca*. Liberibacter has been reported to reduce the lifespan of PoP [19,20]. Laboratory and field studies have shown that PoP adults compared to nymphs, and ACP nymphs rather than adults, are the chief life stages involved in *Ca*. Liberibacter transmission [11,15,16,31]. PoP adults reared on CLso-infected plants were shown to transmit CLso with 100% efficiency, given a one-day inoculation access period (IAP) [31], compared to ACP adults reared on CLas-infected citrus plants, at 6.3% [16]. Until this report, gene expression has not been explored to evaluate potential age-related, phenotypic consequences of *Ca*. Liberibacter infection of adult and nymphal psyllid stages. Understanding the genetic and biochemical mechanisms involved in the interplay between *Ca*. Liberibacter and the different life stages of the psyllid host is expected to provide clues about host-parasite co-evolution in light of the need for host factors to be co-opted by Liberibacter essential for invasion-virulence, infection establishment and multiplication, systemic circulation, and Liberibacter persistence such that the bacterium is transmitted to its plant host.

**Figure 4:**
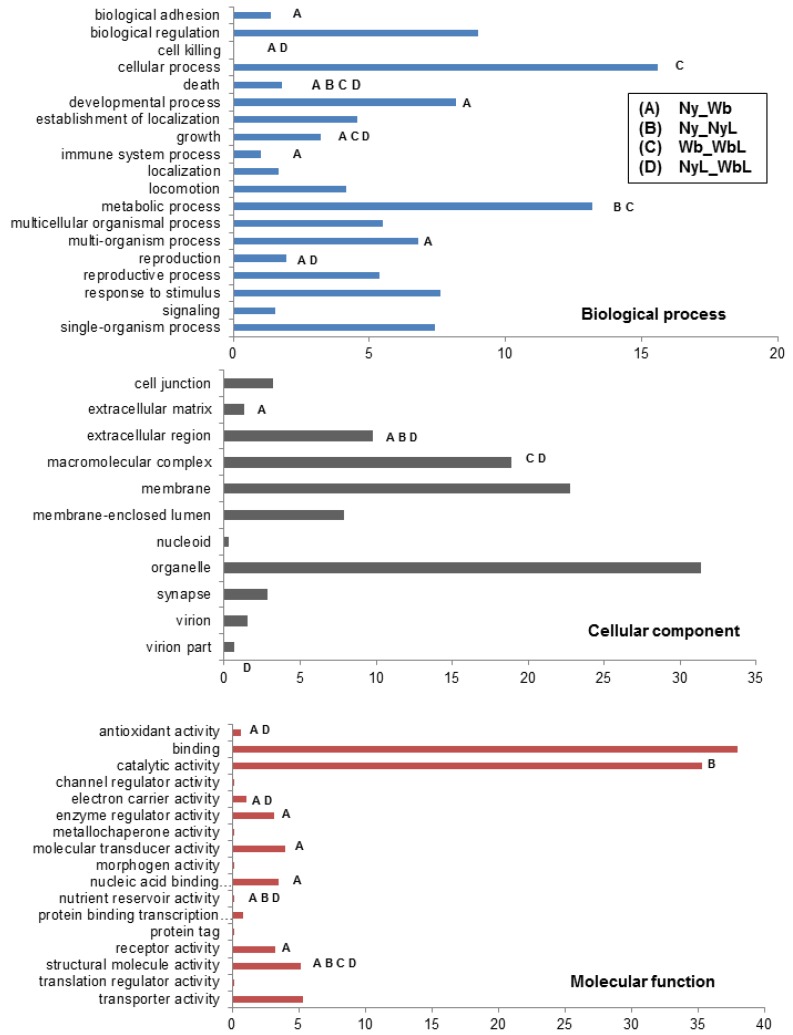
Differential expression of potato psyllid nymph and adult contigs within Gene Ontology categories. The potato psyllid transcripts could be assigned to 46 Gene Ontology functional classes. The x-axis indicates the percentage of contigs assigned to each class. The goseq statistical package was used to determine the assignments that contained an over-representation of differentially expressed contigs between treatments and indicated by a letter: pair wise comparison between PoP nymphs and adults (Ny/Wb; A), infected and uninfected nymphs (Ny/Ny; B), infected and uninfected adults (Wb/WbL; C) and infected nymphs and adults (NyL/WbL; D).

The Gene Ontology (GO) functional classification system [57] was used to classify PoP psyllid contigs for and among the different treatments (psyllid species, adults and nymphs, Liberibacter infection or Liberibacter-free). Forty-seven percent of PoP contigs (38,988) were represented by 50 functional groups (GO levels 1–2) (Figure 4). Among these 13,143 contigs were assigned to the biological process category, 11,969 were affiliated with cellular process, and 13,876 were assigned to molecular function. The ACP contigs (92%) were similarly distributed among the same three GO function categories [40]. Among the 47 PoP level 2 GO categories, 18 (38%) were identified as containing a significant number of differentially transcripts from comparisons of either uninfected adult *versus* nymph (Ny_Wb) or of infected adult *versus* nymph (NyL_WbL), underscoring the importance of life stage to the gene expression profile (Figure 4).

Here, significantly expressed transcripts were defined as those that had a *p*-value cut-off of 0.05 (using edgeR scoring [44]; Supplementary Tables S2–S5). Of 82,224 PoP transcripts, 3313 and 3533 transcripts were differentially expressed at 2-fold or greater with *p-*values < 0.05 in response to CLso-infection in adults and nymphs, respectively (Supplementary Tables S2 and S3). Based on the main level 1 GO category assignments, most contigs had less than a 2-fold change in expression (Table 1). Approximately the same number of differentially expressed ACP and PoP transcripts was up- or down-regulated, however, the majority of down-regulated contigs belonged to PoP, while the up-regulated contigs were from ACP (Table 1). The differentially expressed contigs exhibiting a 2-fold or greater change in expression were correlated with Liberibacter infection, and/or to adult or nymph life stage, with life stage-associated altered expression being the most abundant (Table 1).

**Table 1:** Overall impact of *Candidatus* Liberibacter-infection on potato and Asian citrus psyllid adult and nymph contigs assigned to the level one Gene Ontology categories.

Expression Response	Biological Process				Cellular Process				Molecular Function			
	PoP		ACP		PoP		ACP		PoP		ACP	
	Adults	Nymphs	Adults	Nymphs	Adults	Nymphs	Adults	Nymphs	Adults	Nymphs	Adults	Nymphs
Up-regulated	7%	4%	14%	28%	7%	3%	14%	29%	9%	4%	14%	28%
Down-regulated	7%	14%	14%	11%	6%	14%	13%	11%	8%	17%	14%	11%
Unchanged	86%	82%	72%	61%	87%	83%	73%	60%	83%	79%	72%	61%

The most highly, differentially expressed PoP contigs that have predicted relevance to psyllid host-Liberibacter biological interactions were those related to biological adhesion, immune system processes, locomotion, and reproduction. Among these (Table 2) was a peritrophin-1, a chitin-binding protein found in the peritrophic matrix of insects, an important epithelial barrier to pathogen/parasite invasion [58,59]. Peritrophin-1 expression was found to be down regulated in CLso-infected nymphs (Supplementary Table S3), perhaps having the effect of increased vulnerability to invasion, compared to adults, resulting in the observation of lowered survivability of CLso-infected compared to -uninfected nymphs [19,20]. Further, among the 25 PoP contigs that exhibited the greatest differential expression in response to CLso infection (Table 2), 16% (4/25) were heat-shock-like proteins, and only a single such contig was found for PoP adults indicating that very different stress responses are stimulated by CLso infection in the psyllid life stages. In a parallel study of ACP adults and nymphs subjected to CLas infection, psyllid heat shock proteins were expressed to a greater level in the adults than in the immature stadia (Vyas *et al.* submitted 2014; see Supplementary Table S4). Taken together, we observed an inverse pattern of heat shock protein induction in adults and nymphs for both psyllid-Liberibacter pathogen complexes, a pattern that may be linked with reported stage-specific vector competence, *i.e.*, PoP adults and ACP nymphs, suggesting that the induction of heat shock proteins may be involved in CLso/CLas virulence, in that adults over nymphs are differentially susceptible to the pathogen. The intraspecies differences in life stage most influenced by Liberibacter infection provide robust evidence of distinct host- and Liberibacter-specific gene regulation patterns and underscore the importance of host life stage to Liberibacter parasitic strategies. These observations are consistent with the differential patterns of susceptibility of adults and nymphs to Liberibacter infection [11,16], and of unique host-parasite co-evolutionary relationships that characterize parasite adaptation, invasion, establishment and systemic infection of the different Liberibacter species [60,61].

#### 2.2.1. Adhesion-Related Transcripts

Approximately 15% of all PoP contigs assigned to the biological adhesion category were significantly (*p* < 0.05), differentially expressed in adults and nymphs, with fold change ratios ranging from 3 to 300 times. Several highly differentially expressed contigs were annotated to chitin and cuticle proteins. Both of these protein types are known to be essential for insect growth and morphogenesis, and are involved in processes that modify chitin-containing structures [62]. The altered expression of adhesion-related contigs in PoP adults and nymphs could reflect an involvement in anatomical rearrangements during larval morphogenesis and adult gut epithelial renewal. Feasibly, proteins essential for adhesion-related physiological processes could influence transmission competency in adults and/or nymphs based on the expected requirement by Liberibacter to attach and persist.

For example, *Xyella fastidiosa* requires chitin localized in the mouthparts to colonize its homopteran insect host and vector [63]. The fastidious bacterial plant pathogen transmitted by *X. fastidios*a differs from *Ca*. Liberibacter by being non-propagative and non-circulative in the vector, and, it systemically invades the plant host vascular system, mainly in the xylem. Because chitinolytic enzymes also can exhibit antibacterial properties [64] the “minimal” representation of immune pathway genes that encode antimicrobial peptides in insect vectors of fastidious, bacterial plant pathogens could reflect a protective mechanism of some kind, and if so could be involved in protection of bacterial endosymbionts [25,26,27,28,29,30] or perhaps *Wolbachia*. Indeed this endochitinase was up-regulated 30-fold in Liberibacter-infected PoP nymphs (Supplementary Table S3).

Annexins are important for membrane organization and molecular trafficking [65]. Recent studies have demonstrated a role for an annexin in endocytosis-mediated-invasion [66] of insect peritrophic membranes and midgut epithelial cells by *Plasmodium* [67], resulting in loss of protection against parasitic invasion. In PoP an annexin-B9 transcript was up regulated 11-fold in the CLso-infected, compared to uninfected, nymphs and adults (Supplementary Tables S2 and S3), suggesting an unknown role in CLso virulence.

**Table 2:** The differentially expressed contigs for *Candidatus* Liberibacter solanacearum-infected and uninfected potato psyllid nymphs and adults, based on a *p*-value cutoff of 1E-6.

Nymph	Fold Change *	Adult	Fold Change *
Putative salivary protein	657.71	Cuticular protein 100A	49.29
Abdominal-B	91.35	Cuticular protein PpolCPR68	46.07
Cuticle protein 7	77.92	Putative ribosomal protein S13e	−23.42
Heat shock protein 70 A1	−120.68	Aconitate hydratase, mitochondrial	−120.89
Heat shock 70 kDa protein IV	−337.04	Cytochrome b561	17.63
Ubiquitin	−256.48	Senescence-associated protein, putative	19.59
60S ribosomal protein L5	−175.71	ATP synthase subunit alpha, mitochondrial	−109.49
Heat shock protein 70	−67.87	Arginine kinase	30.16
Putative ribosomal protein S13e	−69.98	60S ribosomal protein L5	−20.22
Probable RNA-directed DNA polymerase from transposon BS	−61.87	Endonuclease-reverse transcriptase	−96.94
Putative reverse transcriptase	−47.65	Ubiquitin	−25.09
FixR	26.65	Cathepsin D	−20.77
Translationally-controlled tumor protein homolog	−92.8	Reverse transcriptase-like protein	−27.8
Cathepsin B preproprotein-like protein	24.68	Meiotic recombination protein SPO11, putative	−21.61
Pyrroline-5-carboxylate dehydrogenase	−148.22	Protein painting of fourth	12.88
Vitellogenin	−40.34	Elongation factor 2	−23.81
Reverse transcriptase-like protein	−83.13	Phosphate carrier protein, mitochondrial	17.1
Putative small heat shock protein	−23.9	Retrovirus-related Pol polyprotein from transposon opus	11.87
SARTTc1 ORF2 protein	−29.89	Probable RNA-directed DNA polymerase from transposon BS	10.07
Tubulin alpha chain	−121.15	Cuticular protein 97Ea	9.96
Meiotic recombination protein SPO11, putative	−42.53	Enolase	−17.11
ATP synthase subunit alpha, mitochondrial	−25.5	F-element protein	−19.39
1-phosphatidylinositol-4,5-bisphosphate phosphodiesterase epsilon-1	11.15	Putative small heat shock protein	−15.97
Peritrophin-1, putative	−39.63	ATP-dependent RNA helicase p62	−14.72
Insecticide resistance protein CYP4G70	−96.66	Cathepsin B preproprotein-like protein	−11.99

* Fold change value based on infected *versus* uninfected comparisons.

#### 2.2.2. Immune System-Related Transcripts

**Figure 5:**
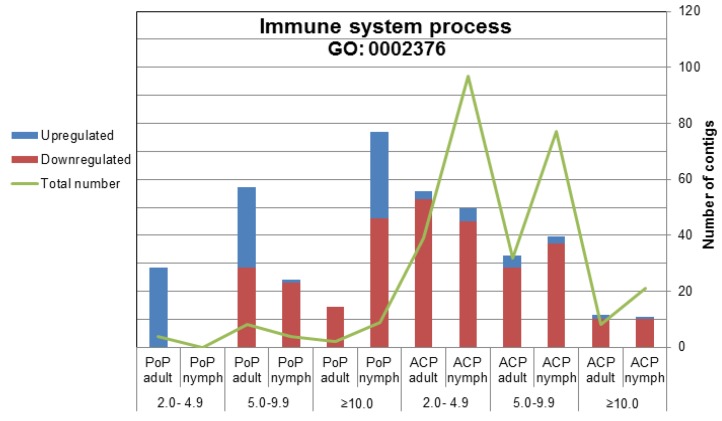
Differential expression of immune-related contigs from the adult and nymphal stages of the potato and Asian citrus psyllid. The significantly differentially expressed potato and Asian citrus psyllid contig (*p* > 0.05; Ny/NyL, Wb/WbL) assignments to the Gene Ontology category Immune System Process (GO:0002376). Blue portion of bar indicates percentage of contigs that were up-regulated (*i.e.*, higher in infected psyllids) in response to *Ca*. Liberibacter infection. Red portion of bar indicates the percentage of contigs that were down-regulated (*i.e.*, lower in infected psyllids) in response to *Ca*. Liberibacter infection. The right x-axis denoted with green line indicates the number of contigs.

The differential expression profiles together with gene ontological categorization of the PoP and ACP contigs revealed striking differences between the psyllid species. Among the total DE transcripts fewer than 1% of PoP contigs were immune-related, whereas, ACP had approximately 5%, and greater than 90% of these ACP immune-related DE contigs were down-regulated, compared to only 50% for PoP (Figure 5). Further, a greater number of immune-related contigs were differentially expressed in ACP nymphs compared to adults, and 92% were down regulated, pointing to a “weak” immune response by ACP nymphs to CLas invasion. Consistent with the above profiles, 62% of the immune-related DE contigs were down regulated in PoP nymphs, compared to 43% for adults, indicating a “weaker” immune response by PoP nymphs. Perhaps this suggests that CLas/CLso infection at the nymphal stage is a requirement for efficient adult-mediated bacterial transmission.

The innate immune system of some insects utilizes cell-mediated and humoral responses to pathogen invasion and attack [68], among which involve the synthesis of antimicrobial peptides usually through Toll and Immunodeficiency (IMD) pathways. These responses can result in melanin formation, and the stimulation of phagocytosis, and encapsulation. Psyllid counter attack to Liberibacter invasion and parasitism likely requires a competent immune system, while also balancing against hyper-immune expression that can potentially cause harm to the host and/or to its beneficial endosymbionts [69]. Indeed many bacteria, including *Ca*. Liberibacter form complex biofilms that provide protection from immune recognition [70,71,72], and studies in our lab have shown that CLso develops biofilms in and on PoP guts prior to circulation in the blood to reach the salivary glands [18].

##### 2.2.2.1. IMD Pathway

The (level 2) gene ontology-classified transcripts identified 907 PoP and 1226 ACP immune response transcripts, corroborating a previous report that the IMD pathway is suppressed in the potato psyllid [73]. Among these, two transcripts shared similarity with a caspase-1 protein (Supplementary Table S6). However, only caspase-8 homologs (*dredd*) are known to be components integral to the IMD pathway [74]. Caspases belong to a family of enzymes identified as effectors of apoptosis. Interestingly, two other transcripts shared similarity with an inhibitor of apoptosis-1, (IAP-1) and IAP-2 genes in the PoP transcriptome, pointing to inhibitory activity potentially directed at the IMD pathway (Supplementary Table S6). The IMD pathway responds to Gram-negative bacterial infection, however, it may not be capable of combatting Gram-negative bacteria like Liberibacter in a reduced state.

##### 2.2.2.2. Toll Pathway

The Toll pathway is induced by gram-positive bacteria and fungi, and could be involved indirectly in the PoP-CLso pathosystem, perhaps through cell-mediated phagocytosis [75]. Transcripts that shared homology with all of the major protein components of the Toll pathway were identified in the psyllid transcriptome, some of which are *Toll, pelle, tube, cactus, dorsal, spätzle*, and *MyD88* homologs. The S*pätzle* homologs were significantly (*p* < 0.05) over expressed in uninfected nymphs compared to uninfected adults (Supplementary Table S4). However, the serine proteases required for *spätzle* activation *nudel*, *gd* (gastrulation defective), *snake*, and *easter* [76] were significantly down-regulated (*p* < 0.05) by 4- to 30-fold in the uninfected nymphs compared to uninfected adults (Supplementary Table S4), suggesting that nymphs are capable of a robust anti-bacterial response to gram-positive and fungal intruders, but later, may be less effective in combatting further invasion.

##### 2.2.2.3. Phagocytosis

Profiles of the ACP and PoP immune-related contigs (significantly DE) responding to Liberibacter infection (Figure 5, Supplementary Table S6) reveal a number of proteins involved in phagocytosis. Classical phagocytosis is affiliated with pathogen-related innate immune responses, and serves as a first line of defense, as well as in tissue homeostasis and remodeling [77]. However, *Rickettsia* uses phagocytosis for epithelial cell entry [78]. Hijacking host factors to facilitate invasion is well documented among pathogenic bacteria [79,80], and underscores the potential for CLas/CLso exploitation of phagocytosis and possibly other exo-endocytic pathways of great interest. In this light, the expression of a diaphanous-like contig was found to be significantly reduced (*p* = 0.0012), at >19-fold in CLso infected PoP adults compared to nymphs (Supplementary Table S6). In a well-known pathosystem, the knockdown of diaphanous-1 (Dia1) interfered with Rac-1 activation, which is required to trigger phagocytosis [81,82]. Additionally, Rac-1 antagonism by a bacterial pathogen to maintain host cell viability that provides a permissive environment for the bacteria to replicate or evade host defenses has been shown [83]. The down-regulation of Rac-1 could result in less efficient clearing of CLso from nymphs, compared to adults, and may implicate phagocytosis-like involvement in CLso invasion.

Two phagocytosis-inducing contigs; GTP-binding Di-Ras [84,85] and vacuolar protein sorting 16B (Vps16B) [86] were significantly up-regulated (*p* < 0.05) 5-fold in CLas-infected ACP adults compared to CLas-infected nymphs (Supplementary Table S6). Di-Ras, which stands for a distinct subgroup of Ras family proteins, are known to induce large cellular vacuolation in the host [84], a potentially critical step in pathogenesis. For example, the vacuolating cytotoxin (VacA) of *Helicobacter pyloris* strains aid in uptake of *H. pylori* outer membrane vesicles (OMV) by host epithelial tissues [87]. Similarly, CLas may enhance ACP Di-Ras activity, in the less efficient host life stage, to promote epithelial cell death and/or apoptosis and increase cellular vacuolation to better facilitate dissemination in the psyllid.

Another phagocytosis related transcript (or contig) had similarity to a 1-phosphatidylinositol-4,5-bisphosphate phosphodiesterase epsilon-1 (PLCE-1) gene was 11-fold upregulated in CLso-infected PoP nymphs, but 4-fold down-regulated in CLas-infected ACP nymphs (Supplementary Table S6). PLCE-1 is a phosphoinositide-specific phospholipase C, an enzyme that enhances phagocytosis [88,89]. The opposing patterns of up and down regulation of PLCE-1 in PoP and ACP nymphs, respectively, suggest that the association of phagocytosis-related contigs with Liberibacter infection is important and warrants further investigation. For example, in ACP nymphs, the down-regulation of phagocytosis related transcripts may weaken the initial immune response. This could explain the greater susceptibility of this life stage to CLas invasion [16] and the higher rate of transmission compared to adults that were not exposed to CLas as nymphal instars [11].

#### 2.2.3. Metabolic-Related Transcripts

The effect of Liberibacter on psyllid metabolic pathways was made evident from comparisons (*p* < 10^−5^) of CLso-infected and uninfected PoP adults (Supplementary Table S8). This observation is not surprising since many bacteria have limited metabolic capabilities and rely on their host for energy precursors. For example, the branched chain amino acid biosynthetic pathways (to isoleucine, leucine and valine) are lacking in the CLso genome [90] and this may lead to host nutrient scavenging. Acyl-CoA dehydrogenases are mitochondrial flavoenzymes involved in fatty acid and chain amino-acid metabolism [91,92]. The greater than 19-fold reduction of an Acyl-CoA dehydrogenase-like contig in a psyllid may be associated with CLso’s inability to carry out *de novo* synthesis of essential amino acids [90] and the psyllid response to prevent leaching thus suppressing bacterial growth.

Two contigs essential in the glycolysis and gluconeogenesis metabolic pathways were significantly (*p* < 0.05) down regulated in CLso infected nymphs compared to the uninfected (Supplementary Table S7). The first, an enolase-like contig was more than 80-fold decreased in CLso-infected nymphs. Enolases mediate immune evasion by degrading host tissues to prevent invasion [93] making its reduced expression potentially detrimental to CLso. The second gene, a phosphoglucomutase-like contig was 20-fold reduced (Supplementary Table S7), is implicated in lipopolysaccharide biosynthesis and biofilm formation [94,95], suggesting the psyllid down-regulates its glycolysis and gluconeogenesis to suppress bacterial invasion and multiplication.

##### Iron Metabolism

Iron regulation is known to be essential for successful entomopathogenic- and symbiotic bacterial interactions with insect host [96,97,98]. Data herein suggest that this scheme holds true for CLso as well, in that more than a third of the GO assignments that contained a significant number of differentially expressed contigs (*p* < 1 × 10^−10^) were involved in functions relating to ion regulation, with iron being the predominant ion (Supplementary Table S9). Ferritin is an iron storage and transport protein important for antioxidation and detoxification [99], and the iron transporter, transferrin, has antioxidant properties to combat invasion by pathogens and parasites [100]. Both of these contigs were significantly up regulated in CLso-infected adults and less so in CLso-infected nymphs (Supplementary Table S10), indicating that iron is essential for CLso pathogenesis of PoP, and therefore a potentially lucrative target for RNAi.

### 2.3. Orthologs in the Potato and Asian Citrus Psyllid Transcriptomes

We explored orthologs, or groups of genes that are similar in sequence, structure, domain architecture and function [101] to identify relevant biological functions central to ACP and PoP, as related psyllid species. Also of interest were species specific differences in physiological and metabolic adaptation that could illuminate evolutionary and functional conservation of host-pathogen interactions in a circulative, propagative mode of transmission. The ACP and PoP transcriptomes include all contigs in CLso/CLas-infected and -uninfected adults and nymphs that are potentially involved in host-pathogen interactions. Coding regions in the PoP and ACP contigs were identified using ESTscan [102], available in the TCW, and these were submitted OrthoMCL clustering [103], also available in the TCW, to identify orthologous genes (contigs).

**Figure 6:**
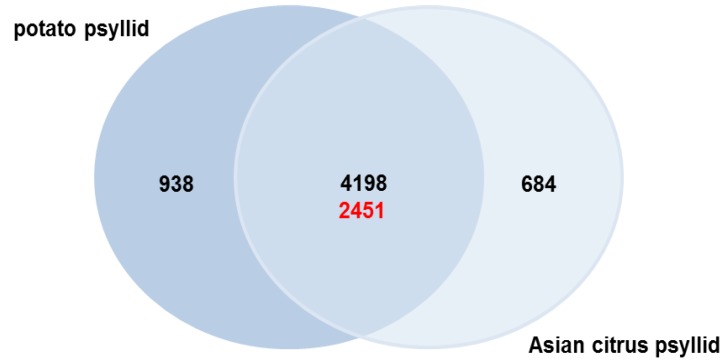
Classification of Asian citrus and potato psyllid contigs into orthologous groups and clustering of transcripts. (A) Numbers of orthologous groups detected across the two species with OrthoMCL. The one-taxon groups (1 × N) are unique to each psyllid species. Two-taxon are found in both and are categorized as single copy (2 × 1; red letters) and multicopy (2 × N; black letters).

Using this approach, it was possible to identify coding regions in 57% and 41% of the ACP and PoP contigs, respectively, totaling 60,085 coding sequences (CDS) of which 67% were assignable to 8721 orthologous groups or clusters (Supplementary Table S11) using OrthoMCL default parameters (1.5 inflation value; 103). Clusters ranged from 2 to 285 CDSs in size those containing 5 or more clusters predominating (Figure 6). The OrthoMCL-defined orthologous groups include one-taxon multicopy (1 × N), two-taxon single copy (2 × 1), and two-taxon multicopy (2 × N) groups representing paralogous (one-taxon) and orthologous (two-taxon) gene families.

**Figure 7:**
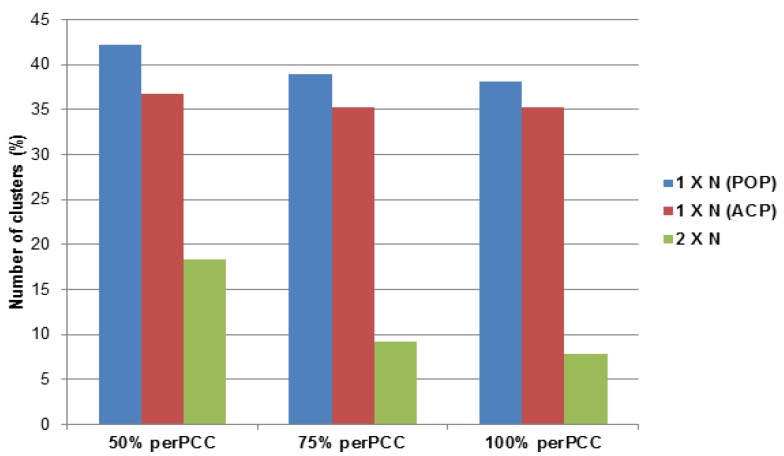
Analysis of OrthoMCL multicopy cluster member expression. Expression (*i.e.*, RPKM-reads per kilobase per million) values of each sequence pair in a cluster were compared for their Pearson Correlation Coefficient (PCC). Graph shows percent of pairs per cluster with PCC values ≥ 0.8 (y-axis) *versus* percentage of total number of clusters in the category (x-axis). Single taxon groups (1 × N) contained more clusters of uniformly expressed contigs (≥50% perPCC in comparison to two-taxon groups (2 × N).

Also, the RPKM value of each sequence (CDS) pair per cluster was compared using the Pearson Correlation Coefficient (PCC) [104], an average value was calculated for each cluster (perPCC) so that they could be filtered based on percentages of pairs having a PCC value of ≥0.8. The perPCC [105] value provides a statistical indication of the extent to which the contig expression is synchronized within a cluster. The results indicated that ACP and PoP contig expression profiles were distinct (Figure 7), despite sharing a large number of genes in common. This might have been expected given distinct host range, different environmental niche, and transmission phenotypes associated with the adult and nymph life stages. The perPCC values ranged from 35% to 42% within the single-taxon groups, but decreased to 8%–18% when both PoP and ACP were included (two-taxon; Figure 7).

#### 2.3.1. Potato Psyllid Paralogous Gene Families

In PoP, 15% of the CDSs were assigned to single-taxon clusters. One such set was containing four members coding for *trans*-sialidase-like proteins. Sialidases are glycosylhydrolases that can contribute to the provision of free sialic acid [106]. Sialic acid is synthesized *de novo* by bacteria or scavenged from the host as a protective covering, providing protection from innate host immunity or used as a nutrient source [107]. Expression of these contigs was at near steady state levels in PoP adults, but was more than 3-fold down-regulated in nymphs in response to Liberibacter infection (Supplementary Table S12). The reduced expression in infected nymphs could be the result of the immature psyllid host’s efforts to reduce pathogen presence by eliminating protective or nutritive resources potentially vital for CLso survival. Another group shared amino acid sequence similarity to a hemolysin-E gene (*hlyE*). These hemolysin-E like contigs are present in both CLso-infected and-uninfected psyllids, indicating that they are of psyllid origin, or they represent carry over during isolation procedures of transcripts for PoP endosymbionts. HlyE is a novel pore-forming toxin produced by *Escherichia coli,* is distinct from the well-characterized pore-forming *E. coli* hemolysins [108], and in the presence of lipids forms transmembrane pores in small intestine epithelial cells [109]. A similar pore-forming toxin was characterized from the kissing bug, *Triatoma infestans,* which showed both antimicrobial and lytic activity towards *Trypanosoma cruzi* and human cells [110]. Only one of the nine HylE-like contigs in this group showed significant expression changes with a 14-fold increase in expression in infected compared to uninfected PoP adults (Supplementary Table S12), likely indicating an antibacterial role. However, CLso may utilize the pore-forming activity to invade or to escape.

#### 2.3.2. Asian Citrus Psyllid Paralogous Gene Families

In ACP, 9% of the CDSs were assigned to single-taxon clusters. Several may be putatively involved in host-pathogen interactions. This includes a three-member group of contigs with similarity to the haematopoietic transcription factor *serpent*, a known regulator of insect immunity [111]. These were detected in both CLas-infected and -uninfected ACP and overall expression was reduced in CLas-infected ACP adults and nymphs (Supplementary Table S11).

Cluster analysis also showed that adult and nymph ACP have an additional calpain family member, compared to PoP adults and nymph (Supplementary Table S11). Calpains are a large family of calcium-regulated cytosolic cysteine peptidases involved in many cellular processes, including the rearrangement of cytoskeletal proteins, signal transduction pathways, and in apoptosis by catalyzing the controlled proteolysis of targeted proteins [112].

#### 2.3.3. Asian Citrus and Potato Psyllid Orthogolous Gene Families

Even though ACP is oligophagous [113] it feeds primarily on species in the Rutaceae, and transmits three citrus-infecting *Ca*. Liberibacter species, while PoP is polyphagous on a wide range of solanaceous as well as other plant species [1], and specifically transmits CLso, the majority (91% and 85% for ACP and PoP, respectively) of the CDSs identified were detected in both psyllid species (at 2 × 1 and 2 × N), suggesting that these two psyllid species harboring different Liberibacter are nonetheless similar at the amino acid (protein) level.

To identify gene families common to both psyllids with potentially conserved biological relevance to psyllid biology and/or Liberibacter transmission, filtering was also carried out using more stringent constraints than those set by the default parameters of the software, requiring at least 80% overlap and 80% sequence similarity among the OrthoMCL-defined groups. The analysis yielded 4836 orthologous groups that contained 2 to 43 CDSs, and reduced the number of 2 × N groups by ~90% to 458 (Supplementary Table S13) due to the exclusion of less functionally similar orthologs. Further, only 9% of the clusters in the 2 × N groups had PCC values ≥0.8 (Table 3), underscoring the variability of expression profiles of the two psyllids. In light of this, identifying the commonalities shared between the ACP and PoP could be instrumental in illuminating conserved host-pathogen proteins and mechanisms involved in circulative, propagative transmission.

**Table 3:** List of top two-taxon multicopy OrthoMCL groups found in the potato and Asian citrus psyllid transcriptomes, including group size, description (UniProt), and Pearson Correlation Coefficient values.

Cluster ID	Size	Description	perPCC
TR_000786	3	3-hydroxy-3-methylglutaryl-coenzyme A reductase	100
TR_003785	3	Abc transporter	100
TR_000835	3	Atlastin	100
TR_001975	3	Calmodulin	100
TR_001355	3	cAMP-dependent protein kinase catalytic subunit, putative	100
TR_000708	4	Chitin synthase 1 variant b	100
TR_000290	3	Cleavage and polyadenylation specificity factor subunit 1	100
TR_001473	3	Cytochrome P450, putative	100
TR_000343	4	DNA replication licensing factor MCM4	100
TR_000489	3	DNA-binding protein Ewg, putative	100
TR_001764	3	Dual oxidase maturation factor 1	100
TR_000565	3	Dynamin-like 120 kDa protein, mitochondrial	100
TR_000908	3	E78 nuclear receptor	100
TR_000324	3	EH domain-binding protein 1	100
TR_000905	3	F-box only protein, putative	100
TR_000639	3	Forkhead box protein K1	100
TR_000486	3	Hedgehog protein	100
TR_000141	3	Inactive ubiquitin carboxyl-terminal hydrolase 54	100
TR_000345	3	Kinesin-like protein KIF21B	100
TR_000433	3	Merlin	100
TR_001523	3	Mitogen-activated protein kinase ERK-A, putative	100
TR_000402	3	Phosphatidylserine synthase 1	100
TR_002280	4	Probable aconitate hydratase, mitochondrial	100
TR_000043	3	Probable phosphorylase b kinase regulatory subunit alpha	100
TR_000068	4	Probable pre-mRNA-splicing factor ATP-dependent RNA helicase mog-5	100
TR_000493	3	Protein turtle	100
TR_000691	3	Protein Wnt	100
TR_000597	3	Proton-coupled amino acid transporter 4	100
TR_001033	3	Putative AMP dependent CoA ligase	100
TR_000510	3	Pyruvate dehydrogenase E1 component subunit beta, mitochondrial	100
TR_000027	3	SH3 and multiple ankyrin repeat domains protein 3	100
TR_001667	3	Shc transforming protein, putative	100
TR_001186	3	Techylectin-5B	100
TR_000532	3	Transient receptor potential cation channel trpm	100
TR_000454	3	Triacylglycerol lipase, pancreatic, putative	100
TR_000971	4	Actin-binding protein IPP	83.33
TR_001171	4	Putative GPCR-type octopamine beta receptor (Fragment)	83.33
TR_000754	4	Sodium/hydrogen exchanger	83.33
TR_000042	4	Tyrosine-protein kinase transforming protein FPS, putative	83.33
TR_000375	5	Ephrin receptor	80

Nipah and Hendra viruses (*Henipavirus*) enter mammalian host endothelial cells by attachment to the ephrin-B2 and/or -B3 receptors [114]. A five-member 2 × N group sharing an interesting similarity with the Ephrin-like receptors, were down-regulated in response to CLas and CLso, suggesting these genes could be involved in bacterial invasion of psyllid gut and/or salivary gland tissues. Another 2 × N group that contained three-members showed similarity with transient receptor-potential proteins (Supplementary Table S13), which are a multigene superfamily of integral membrane ion channel proteins that participate in diverse physiological processes, including sensing stimuli and ion homeostasis [115]. A three member 2 × N group coding techylectin-like immune proteins (Supplementary Table S13), were differentially expressed in CLas/CLso infected adults and nymphs. These kinds of proteins are known to have a role in phagocytosis and participate either by responding to immune defenses [116,117], and/or in host cell membrane invasion. Based on the *in silico* identification of several phagocytosis-related proteins in the ACP and PoP transcriptomes, Liberibacter may interact with them as well as techylectin-like proteins as a common mechanism for psyllid cell entry during invasion. Two additional contigs identified by cluster analysis with a possible role in host-pathogen interactions were phosphatidylserine synthase 1 and triacylglycerol lipase (three members, each) (Supplementary Table S13), enzymes involved in sphingolipid and/or phospholipid metabolism [118,119]. These enzymes are not produced by the majority of bacteria but many use host sphingolipids as cell-surface protectants for evading host immune responses, to facilitate colonization [118], and perhaps in biofilm formation [120].

## 3. Methods

### 3.1. Psyllids

*Ca*. Liberibacter solanacearum (CLso) -infected psyllids were collected from a psyllid-infested tomatoes from a greenhouse in Arizona in 2004 and maintained as a laboratory colony at the University of Arizona since then. CLso-infected psyllids were reared on tomato plants (Roma) under laboratory conditions with temperature of ~24 °C and a photoperiod of 12:12 h (light and dark) and transferred serially to fresh plants approximately every 3–5 weeks. The CLso-free (uninfected) potato psyllids were obtained from Dr. J. Munyaneza. Psyllids were haplotyped as the “central-type” using the cytochrome oxidase I gene as a molecular marker [121] and colonies were routinely checked for CLso presence or absence by PCR using primers that amplify the 16S rRNA gene [3,122]. Adult and nymphs (2–5 instar) were collected so that the most complete whole transcriptome data set could be generated over a complete range of adult and nymph life stages representing complete cohorts minus the first instar, which was too small to obtain near-equal body weight in comparison to the other nymphal instars. Live psyllids were collected from the colonies and subjected to processing by crushing lightly in RNA-free tubes by micro-pestles, followed by the addition of Trizol (Invitrogen, Carlsbad, CA, USA), prior to overnight shipping on dry ice to Washington State University (WSU) where they were stored at −80 °C until total RNA was isolated. The CLas-infected and -uninfected ACP colonies were reared in laboratory cultures maintained on a CLas host (*Citrus* spp.) or a CLas- immune rutaceous plant species. Cultures were reared continuously and serially transferred periodically to the same host species at the University of Florida Citrus Research and Education Center (courtesy, Dr. K.S. Pelz-Stelinski, Lake Alfred, FL, USA) or at the Southwest Florida Research and Education Center (courtesy, Dr. P.A. Stansly, Immokalee, FL, USA). Adults and nymphs (2–5 instar) were collected and processed in the same manner as described above for PoP and the RNA samples were also shipped to WSU where they were stored at −80 °C until use. ACP transcripts used for comparative analyses in this study were obtained in a similar fashion as described below for PoP and in a simultaneously submitted manuscript [40].

### 3.2. RNA Isolation and Quality Control

Total RNA was isolated from 225 to 250 adult or nymph psyllids either infected or uninfected with *Candidatus* Liberibacter solanacearum. Previously crushed psyllid samples were supplied in 1 mL Trizol each. For RNA extraction, 0.3 mL chloroform were added to 1 mL Trizol homogenate, followed by vigorous sample shaking for 30 s, and incubation for 3 min at room temperature. The samples were centrifuged at 12,000 × g for 15 min at 4 °C to separate the organic from the aqueous phase. The aqueous phase (200–250 μL) was transferred to a new, sterile RNase-free tube and an equal volume of 100% EtOH was added, with mixing. Samples were further purified using the RNeasy Mini Kit (Qiagen, Valencia, CA, USA) according to the manufacturer’s protocol. The quality and quantity of each RNA sample was assessed using a NanoDrop 2000 Spectrophotometer (Thermo Scientific, Wilmington, DE, USA) with A260:A280 ratio greater than 2.0. RNA integrity was confirmed using an Agilent 2100 Bioanalyzer (Agilent Technologies Inc., Santa Clara, CA, USA) with an RNA Integrity Number (RIN) value greater than or equal to 8.

### 3.3. Library Construction and Illumina Paired-End Sequencing

The poly(A) RNA was isolated from 2 μg of total psyllid RNA from each treatment using magnetic oligo (dT) beads. Following purification, the mRNA was fragmented by zinc treatment at 94 °C for 5 min and reverse-transcribed to synthesize first strand cDNA using SuperScript II reverse transcriptase (Invitrogen) and random primers, followed by second-strand cDNA synthesis. The cDNAs were subjected to end-repair and phosphorylation and the addition of an “A” base to the 3’-end of the blunt phosphorylated DNA fragments. Illumina Paired-End (PE) adapters were ligated to the fragments, as described by Illumina’s Paired-End Sample Preparation Guide (Illumina). A 250 bp ± 25 bp smear containing the cDNA fragments, flanked by Illumina PE adapters, was cut from a 2% agarose gel for downstream enrichment. The cDNA fragments were amplified by PCR Primers PE 1.0 and PE 2.0 (Illumina) that anneal to the ends of the adapters, using the PCR program of 30 s at 98 °C followed by 15 cycles of 10 s at 98 °C, 30 s at 60 °C, 15 s at 72 °C and a final elongation step of 5 min at 72 °C. The products were purified using the QIAquick PCR Purification Kit (Qiagen) to create an Illumina paired-end library for each treatment. Library quality control was performed with a Bioanalyzer DNA 1000 Chip Series II (Agilent). A qPCR method was employed to quantify libraries in advance of generating clusters. The libraries were diluted to a final concentration of 10 nM. The paired-end libraries were applied for cluster generation at a concentration of 10 pM in a flowcell on a cBOT (Illumina). Sequencing was performed with one lane per library of 2 × 54 bp reads from both ends of the fragments on an Illumina Genome Analyzer IIx at the National Center for Genome Resources (NCGR). The complete dataset has been deposited to the Short Read Archive (SRA) at GenBank, under accession PRJNA252003, SRX583042, and SRX583048.

### 3.4. Assembly, Annotation and Comparison of Illumina Sequences

The Illumina reads were cleaned and assembled as described in He *et al.* 2010 [123]. Briefly, the reads were assembled with ABySS [124], the gaps were filled used GapCloser in SOAP [125], the scaffolds merged with Mira [126] and filtered for redundancies with CD-HIT [127], and the reads were aligned *post hoc* to the final contig consensus sequences using Burrows-Wheeler Alignment (BWA) [128].

The resulting transcripts and read counts were loaded into Transcriptome Computational Workbench (TCW) [39], where the transcripts were annotated using an E-value cutoff of 10^−10^ against a subset of the UniProt taxonomic databases [46,47] and the GO terms extracted from the “.dat” files [57]. Based on the TCW, levels of significance of differentially expressed transcripts for individual and those grouped in GO categories were determined by edgeR [44] and goseq [129] analysis, respectively. These results were analyzed with the TCW interactive Java interface, publicly available at www.sohomoptera.org. The results for the figures and tables were created using either the TCW software for single (sTCW) or multiple (mTCW) database comparisons. The results shown in Table 1 were obtained using the “include” and “exclude” filter for the libraries of interest selected with minimum 2-fold up and down option for each level 1 GO category (biological process GO: 0008150, cellular component GO: 0005575 and molecular function GO: 0003674). The sum of up and down changes was subtracted from total number of transcripts and expressed as a percent. The results shown in Table 2 and Supplementary Tables S2–S5 were obtained by using the “include” and “exclude” filter for the libraries of interest selected having the differential expression value cutoff of 0.05. The results summarized in Figure 4 were obtained with the “Basic GO Query” with filters set to view level 2 GOs with a differential expression *p*-value threshold of 0.05 for all libraries. The results Supplementary Tables S6–S10 were obtained by selecting “to show” transcripts with reads from all libraries, and all GO levels, either grouped (Table 3) or not grouped by GOs (Supplementary Tables S6–S10). Significance of differential expression was based on either a *p*-value cutoff of 10^−5^ or 10^−10^, with stringency selection based on the results obtained using each method. The results in Table 3 and Supplementary Tables S12–S13 were obtained within TCW using the “transitive” option for cluster analysis, showing all two-taxon multicopy (2 × N) clusters having at least 80% of pairs having Pearson Correlation Coefficient (PCC) value ≥ 0.8.

The TCW executed the ESTScan software [102] on the transcripts to identify peptides with a minimum cutoff of 30 amino acids, using a training matrix produced from the Hemiptera proteins from GenBank. The TCW was used to execute the OrthoMCL software [103] to compute ortholog clusters from these two peptide sets using an inflation parameter of 1.5 to balance sensitivity with selectivity. The orthologous groups were combined in TCW with the annotation and expression levels from the individual TCW databases. Finally, the majority UniProt hit was assigned to each group. The results in Supplementary Tables S11–S12 were obtained using default parameters, showing all clusters.

### 3.5. Transmission Electron Microscopy

For negative staining, CLso-infected and uninfected adults were vortexed in 5% bleach 10 s, and midguts were extirpated in diethyl pyrocarbonate (DEPC) treated H_2_O. Each was transferred to individual pioloform coated grids containing 20 µL of DEPC H_2_O. Five minutes after gentle mastication with insect pins, tissue fragments were removed, and the hemolymph/water solution was allowed to evaporate to a thin film before the addition of 2.5 µL 4% glutaraldehyde. Specimens were fixed 10 min. One drop (~10 µL) of 2% uranyl acetate was added and allowed to stand for 2 min, followed by blotting with filter paper and air-drying. The grid was air-dried for several hours and examined using a Phillips CM-12 transmission electron microscope operated at 80 kV.

## 4. Conclusions

This manuscript highlights the results of an interdisciplinary research effort that has produced the first annotated, differentially expressed transcriptomes of adult and nymphal stage PoP either free of or harboring CLso. This PoP transcriptome database contains 82,224 contigs, of which 16,496 (20%) could be annotated. The availability of the psyllid (PoP and ACP) annotated transcriptome database integrated with comparative profiling and search tools offers a novel opportunity to exploit new knowledge about host-parasite interactions in this pathosystem. Impressive differences in the expression levels of the same or similar contigs occurred for ACP and PoP adults and nymphs infected or uninfected with Liberibacter, suggesting some extent of host-parasite co-evolution, despite the different host insects and Liberibacter pathogen species involved. It is anticipated that functional studies for protein and gene families common to both psyllid species, now in progress, will lead to discoveries of evolutionarily conserved genes important in host-parasite interactions to enhance our knowledge of psyllid biology, as well as those involved in conserved host-parasite interactions across hemipteran insects.

The linked cluster analysis software facilitates the identification of psyllid species- and life stage-specific proteins, and gene families shared across two psyllid species. The close parasitic relationships only recently recognized between certain psyllid species and the genus *Ca*. Liberibacter will make it possible to consider networks of psyllid proteins from similar and different gene families that interact with Liberibacter proteins to further illuminate co-evolutionary relationships involving both parasitic or symbiotic outcomes.

The *in silico* annotated transcriptomes and differential expression profiling of the different life stages of PoP and ACP harboring CLso have produced a lucrative list of effector targets that may serve as determinants of infection, multiplication, circulation and the acquisition process in two different psyllid-Liberibacter study systems. For example, a putative psyllid annexin- and an ephrin receptor-like contigs were identified that may be required for bacterial invasion. Also this list includes many endocytosis-related genes such as a Vps16B and Di-Ras, initially assumed to be involved in phagocytic psyllid immune responses but may be advantageously used by CLas/CLso to gain entry into its host. Metabolic and nutritionally directed investigations led to the addition of enolase- and transferrin-like psyllid genes to this list, both potentially having an effect on CLas/CLso virulence capabilities. Lastly, the *in silico* identification (this study) and subsequent TEM and RT-PCR validation of expressed flagellar- and pilus-like structures adds target effector(s), which may be involved in bacterial persistence and/or dissemination in psyllids, to this list. Functional validation and biochemical characterization of the remaining most promising effector targets are ongoing, and preliminary data suggests significant advancements in our goal of targeting effectors that interfere with psyllid-mediated circulative, propagative transmission of Liberibacter becoming a reality.

The abundance of predicted protein interactors having putative CLso effectors encoded by PoP and ACP nymphs and adults underscore the apparent abundance of direct and indirect psyllid responses to Liberibacter infection, providing evidence of conserved mechanisms in psyllid-Liberibacter interactions. These proteins are cataloged in linked databases to allow for comparative data mining using the ACP/PoP website, and provide a valuable, user-friendly, comprehensive molecular and bioinformatics resources to advance research in potato and Asian citrus psyllid biology, genetics, and functional genomics.

Of particular interest to our efforts are those effector-interactor pairs with pivotal functions in modulating CLas/CLso-psyllid interactions. Examples of these are psyllid proteins that interact with bacterial effectors to mediate virulence, adherence, entry-invasion, nutritional exploitation, evasion of host immunity, and persistence and circulation in psyllid host tissues. Our hope is that these tools will serve as a relevant starting point to guide RNA-interference strategies for abatement of psyllid-mediated Liberibacter transmission, with an immediate and timely emphasis toward managing citrus greening disease.

## Supplementary Files

Supplementary File 1

## Abbreviations

ACP: Asian citrus psyllid
CLas: *Candidatus* Liberibacter asiaticus
CLso: *Candidatus* Liberibacter solanacearum
GO: Gene Ontology
HLB: Huanglongbing
PCC: Pearson Correlation Coefficient
perPCC: average PCC per cluster
PoP: potato psyllid
RPKM: *Reads per KB per million reads*
ZC: Zebra chip disease
