# Supplementary material for: Comparison of Potato and Asian Citrus Psyllid Adult and Nymph Transcriptomes Identified Vector Transcripts with Potential Involvement in Circulative, Propagative Liberibacter Transmission
Source: Pathogens. 2014 Nov 3;3(4):875–907. doi: 10.3390/pathogens3040875 (PMC4282890; doi:10.3390/pathogens3040875)
Supplement: Supplementary File 1 [file pathogens-03-00875-s001.zip › pathogens-66026-supplementary-final/pathogens-66026-supplementary-revised-layout/pathogens-66026-supplementary-layout.doc]

**Supplementary Material**

**Table S1.** Summary of sequencing, assembly and annotation results of the potato psyllid contigs obtained from whole bodies of adult potato psyllids (Wb), whole bodies of adults infected with *Ca*. Liberibacter solanacearum (WbL), potato psyllid nymphs (Ny), and potato psyllid nymphs infected with *Ca*. L. solanacearum (NyL). Transcripts refer to consensus transcript sequences.

**Table S2.** List and summary of statistics for potato psyllids contigs differentially expressed (*p* < 0.5) between comparison of *Candidatus* Liberibacter-uninfected and -infected adults, including normalized expression values (RPKM), differential expressional values (*p*-value), and UniProt annotation.

**Table S3.** List and summary of statistics for potato psyllids contigs differentially expressed (*p* < 0.5) between comparison of *Candidatus* Liberibacter-uninfected and -infected nymphs, including normalized expression values (RPKM), differential expressional values (*p*-value), and UniProt annotation.

**Table S4.** List and summary of statistics for potato psyllids contigs differentially expressed (*p* < 0.5) between comparison of *Candidatus* Liberibacter uninfected-adults and -nymphs, including normalized expression values (RPKM), differential expressional values (*p*-value), and UniProt annotation.

**Table S5.** List and summary of statistics for potato psyllids contigs differentially expressed (*p* < 0.5) between comparison of *Candidatus* Liberibacter infected-adults and -nymphs, including normalized expression values (RPKM), differential expressional values (*p*-value), and UniProt annotation.

**Table S6.** List and summary of statistics for all potato psyllids contigs assigned to the Immune System Process Gene Ontology category (GO: 0002376), including normalized expression values (RPKM), differential expressional values (*p*-value), and UniProt annotation.

**Table S7**. List and summary of statistics for all potato psyllids contigs assigned to the Metabolic Process Gene Ontology category (GO: 0008152), including normalized expression values (RPKM), differential expressional values (*p*-value), and UniProt annotation.

**Table S8.** Lists of Gene Ontology biological and molecular process categories that contain a significant number of differentially expressed transcripts between *Candidatus* Liberibacter solanacearum-infected and uninfected potato psyllid adult comparisons using a *p-*value cutoff of 1E-5.

**Table S9.** Lists of the Gene Ontology biological and molecular process categories that contain a significant number of differentially expressed contigs between *Candidatus* Liberibacter solanacearum-infected potato psyllid adults and nymphs comparisons using a *p-*value cutoff of 1E-10.

**Table S10.** List and summary of statistics for all potato psyllids contigs assigned to the Iron Ion Homeostasis Gene Ontology category (GO: 0055072), including normalized expression values (RPKM), differential expressional values (p-value), and UniProt annotation.

**Table S11.** List and summary of statistics for all psyllid OrthoMCL-defined clusters using default parameters, including UniProt annotation, total number of members, and members in each psyllid species.

**Table S12.** List and summary of statistics for selected psyllid OrthoMCL-defined clusters, including normalized expression values (RPKM) and UniProt annotation.

**Table S13.** List and summary of statistics for psyllid OrthoMCL-defined clusters with at least 80% overlap and 80% similarity of sequence, including UniProt annotation, total number of members, and members in each psyllid species.
